# Meta-analysis and systematic review: the association between rheumatoid arthritis and the risk of thyroid cancer

**DOI:** 10.3389/fimmu.2026.1769646

**Published:** 2026-06-03

**Authors:** Xue Li, Ruihan Liu, Yan Zhang, Jian Li, Xiaohan Wang, Chun Wang, Xi Yang

**Affiliations:** 1 Department of Ultrasound Medicine, The Shapingba Hospital, Chongqing University (People’s Hospital of Shapingba District, Chongqing), Chongqing, China; 2 Department of Scientific Research, The Shapingba Hospital, Chongqing University (People’s Hospital of Shapingba District, Chongqing), Chongqing, China; 3 Academic Affairs Office, The Shapingba Hospital, Chongqing University (People’s Hospital of Shapingba District, Chongqing), Chongqing, China; 4 Department of Emergency, Chongqing Hospital of The First Affiliated Hospital of Guangzhou University of Chinese Medicine, Chongqing Beibei Hospital of Traditional Chinese Medicine, Chongqing, China

**Keywords:** meta-analysis, rheumatoid arthritis, risk, systematic review, thyroid cancer

## Abstract

**Background:**

Rheumatoid Arthritis (RA), as a common autoimmune disease, not only causes damage to the joints themselves, but also increases the risk of developing other diseases. In recent years, the association between RA and malignant tumors has attracted increasing attention. Most studies have found that RA is associated with site-specific alterations in malignancy risk rather than a uniformly increased risk of all cancers. However, the risk of thyroid cancer in RA patients remains controversial. Different studies even yield contradictory results. Notably, to date, no systematic review or meta-analysis has specifically evaluated this association. Therefore, this meta-analysis aimed to estimate the association between RA and the risk of thyroid cancer, providing evidence for clinical cancer surveillance strategies.

**Methods:**

The search was conducted from database inception to October 2025 in PubMed, EMBASE, and Web of Science databases. Studies that estimated the association between rheumatoid arthritis (RA) and the incidence of thyroid cancer using hazard ratio (HR) or standardized incidence ratio (SIR) and 95% confidence interval (CI) were included. Two researchers independently screened the literature, extracted the data, and evaluated the risk of bias of the included studies. Meta-analysis was performed using RevMan 5.3 software. This meta-analysis was registered on the PROSPERO platform (CRD420251267552).

**Results:**

A total of fourteen cohort studies were included, involving 1,970,165 participants. The meta-analysis indicated that patients with RA have a higher risk of developing thyroid cancer compared to the general population (HR = 1.28, 95%CI: 1.05-1.55, P = 0.01). The subgroup analysis showed that RA in the Chinese population increased the risk of thyroid cancer (HR = 1.79, 95% CI: 1.26-2.54, P = 0.001). There was no association between RA in other populations and the risk of thyroid cancer (HR = 1.01, 95% CI: 0.85-1.21, P = 0.88). RA did not increase the risk of thyroid cancer under parsimonious adjustment (adjusted only for age and/or sex) (HR = 1.03, 95% CI: 0.73-1.44, *P* = 0.88), but was associated with an increased risk of thyroid cancer under extended adjustment (HR = 1.38, 95% CI: 1.09-1.73, *P* = 0.006).

**Conclusions:**

The existing evidence suggests that RA may be associated with the risk of thyroid cancer. Given the limitations of this study, well-designed prospective studies with appropriate confounding controls are needed to further verify this association.

**Systematic review registration:**

https://www.crd.york.ac.uk/prospero/, identifier CRD420251267552.

## 1 Introduction

Rheumatoid arthritis (RA) is a common chronic autoimmune disease that frequently affects middle-aged women between the ages of 40 and 60 (1). In recent years, the global prevalence of RA has been on the rise, and the age at which people fall ill has shown a trend of becoming younger (2). Its characteristic manifestations are chronic inflammation and proliferative lesions of the synovium (3, 4). Most patients with RA need to undergo drug treatment to control their condition (5). However, RA is not only a joint disease but also a systemic disorder linked to an altered risk of site-specific malignancies rather than a uniformly increased cancer risk (6, 7).

Malignant tumors are a leading cause of death in patients with RA (8, 9). Mounting evidence indicates that RA is associated with higher risks of lymphoma and lung cancer, but lower risks of colorectal and breast cancer, showing that malignancy risk varies substantially by cancer type (10, 11). Thyroid cancer is the most common malignant tumor in the endocrine system, and its global incidence rate has been continuously increasing (12). Most thyroid cancers (such as papillary carcinomas) have a favorable prognosis, but some pathological types (such as medullary carcinoma, undifferentiated carcinoma) are highly aggressive and even carry a fatal risk (13). Given shared immune dysregulation and inflammatory mechanisms, thyroid cancer is of particular relevance to RA patients because both diseases are closely linked by autoimmunity, chronic inflammation, and overlapping immune disturbances, and its risk deserves focused investigation (14–17).

To date, studies on the association between RA and thyroid cancer risk have yielded inconsistent and contradictory results. Some investigations reported a higher incidence of thyroid cancer in patients with RA, whereas others detected no significant association. These discrepancies are likely driven by differences in sample size, study population, screening intensity, and confounding adjustment. Because individual studies are underpowered or prone to heterogeneity, a systematic review and meta-analysis is necessary to synthesize available evidence, quantify the overall association, and explore sources of between-study variability.

To date, no meta-analysis has been conducted to clarify the association between RA and thyroid cancer risk. Accordingly, this study aimed to integrate published epidemiological data and use meta-analysis to evaluate the association between RA and the risk of thyroid cancer, providing an important reference for clinical cancer surveillance strategies.

## 2 Methods

This systematic review was conducted in accordance with the Preferred Reporting Items for Systematic Reviews and Meta-Analyses (PRISMA) 2020 statement (18, 19).

### 2.1 Search strategy

Searches were conducted in PubMed, Embase, and Web of Science from the database inception to October 2025, aiming to identify relevant studies that evaluated the association between RA and the risk of thyroid cancer using hazard ratio (HR) or standardized incidence ratio (SIR), along with extraction of their 95% confidence intervals (CI). The search strategy combined controlled vocabulary (MeSH terms in PubMed, Emtree terms in Embase) and free-text keywords using Boolean operators AND and OR. The core search string was constructed as follows: (“Arthritis, Rheumatoid”[MeSH] OR “Rheumatoid Arthritis”[Title/Abstract] OR “RA”[Title/Abstract]) AND (“Thyroid Neoplasms”[MeSH] OR “Thyroid Cancer”[Title/Abstract] OR “Thyroid Carcinoma”[Title/Abstract] OR “Thyroid Tumor”[Title/Abstract] OR “Thyroid Malignancy”[Title/Abstract]). No language or publication date restrictions were applied during the database search to maximize sensitivity. In addition, a manual screening of the reference list for the included studies was conducted to identify other potentially relevant studies. The complete database-specific search strings are provided in the **Supplementary Materials** (**Supplementary Table 1**) for readers requiring exhaustive detail.

### 2.2 Inclusion and exclusion criteria

#### 2.2.1 Inclusion criteria

PECOS Criteria:

P (Population): The exposed group consisted of patients with rheumatoid arthritis (RA), and the control group consisted of non-RA individuals.

E (Exposure): Diagnosis of rheumatoid arthritis (RA).

C (Comparison): Non-RA individuals.

O (Outcome): Risk of thyroid cancer. Thyroid cancer must be naturally occurring during the observation period, and the study must clearly report the HR (hazard ratio) or SIR (standardized incidence ratio) with 95% CI for thyroid cancer occurrence.

S (Study design): Cohort study or case-control study.

#### 2.2.2 Exclusion criteria

1. Duplicate publications.
2. Study types: Case reports, case series, reviews, meta-analyses, comments, letters, or conference abstracts.
3. Full text or key data unavailable (e.g., missing effect size or 95% CI).
4. Irrelevant topic or not investigating the association between RA and thyroid cancer.

### 2.3 Data extraction

Retrieved literature was imported into the Endnote software and then deleted the duplicate ones. Two researchers independently screened the eligible studies. Firstly, irrelevant literature was excluded based on the titles and abstracts. Then, each independently read the full text of each article that might be eligible to determine whether it should be included. When the two researchers had a disagreement, an experienced third researcher confirmed it. Finally, the two researchers independently extracted the data, including the following information: the first author’s name, publication year, study period, country, study design, sample size, sex, mean age at baseline, thyroid cancer diagnostic criteria, adjusted confounding factors, average follow-up period, and HR or SIR, as well as 95% CI.

### 2.4 Risk of bias assessment

This study used the Risk of Bias in Non-Randomized Studies of Exposures (ROBINS-E) to evaluate the risk of bias for the included studies. Two researchers independently evaluated the risk of bias for the studies included. The assessment covered the following seven bias domains: confounding bias, participant selection bias, exposure classification bias, bias due to deviations from intended exposure, missing data bias, outcome measurement bias, and selective reporting bias. Each domain was classified as low risk, moderate risk, or high risk according to the standards. Ultimately, the overall risk of bias judgment for each study was determined based on the lowest rating across all domains. In case of disagreement, a third researcher arbitrated to ensure the consistency of the evaluation conclusion.

### 2.5 Data synthesis and analysis

Meta-analysis was conducted using the RevMan 5.3 software. We included studies that reported different measures of relative risk (HR and SIR). Due to the rare nature of thyroid cancer in the general population (low absolute risk), the rare disease assumption holds, under which both HR and SIR can be interpreted as comparable relative risks. Previous meta-analyses on cancer risk have successfully pooled HR and SIR under this assumption (20–23). To ensure statistical power, we pooled all estimates as HR. A sensitivity analysis using only HR-reported studies was conducted to assess the robustness of this decision. If the heterogeneity test showed that P ≥ 0.1 and I² ≤ 50%, a fixed-effect model was used for the analysis. If the heterogeneity test showed that P < 0.1 and I²> 50%, a random-effect model was used for the pooled analysis. Subgroup analysis was conducted based on country (China vs others) and level of covariate adjustment. For the latter, we classified studies into two categories: (1) Parsimonious adjustment: studies that adjusted only for age and/or sex. (2) Extended adjustment: studies that adjusted for age and sex plus at least one additional covariate (e.g., BMI, smoking, alcohol intake, comorbidities, or socioeconomic factors). This classification was chosen to explore whether more rigorous confounding control influences the pooled estimate, rather than using an arbitrary numerical cutoff. Sensitivity analysis was performed using the method of exclusion to assess the robustness of the pooled effect size. The symmetry of the funnel plot was evaluated, and Begg’s and Egger’s tests were used to assess publication bias. All statistical analyses were conducted using Stata 14.0.

## 3 Results

### 3.1 Study characteristics

**Figure 1:**
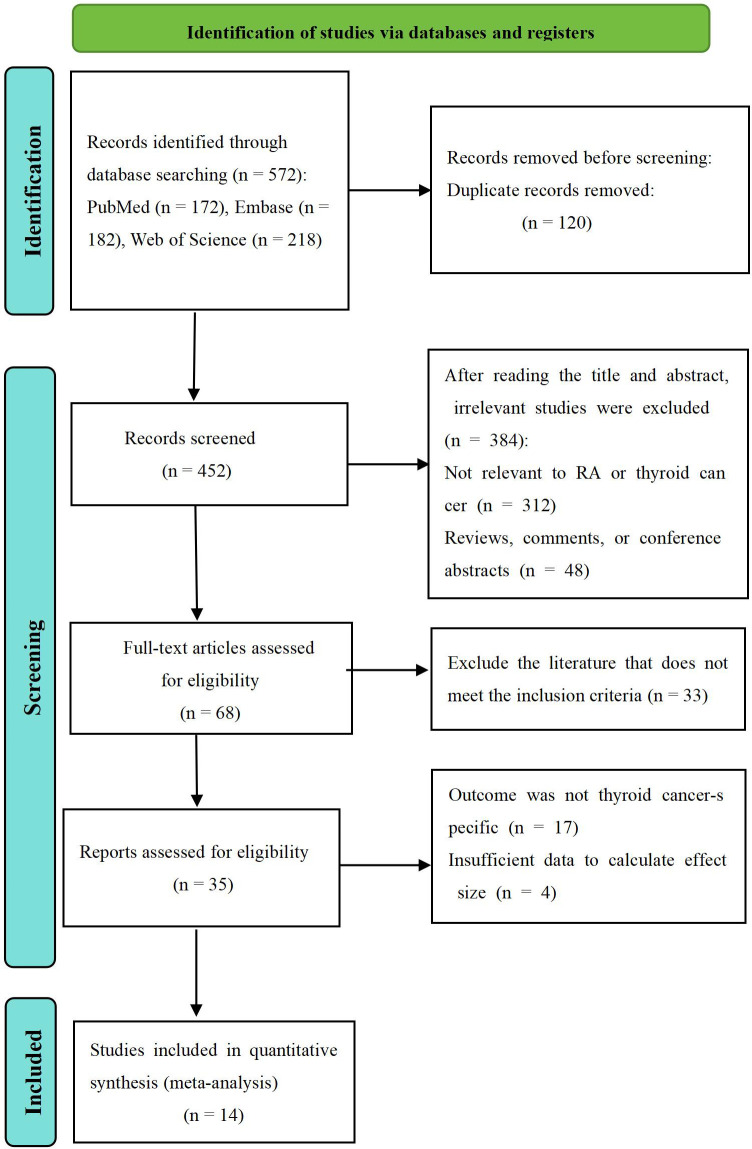
Flowchart of database search and study inclusion.

Through database search and citation screening, a total of 572 potentially relevant records were initially identified from PubMed (n = 172), Embase (n = 182), and Web of Science (n = 218). No additional records were identified through reference list screening. After removing 120 duplicate records, 452 remaining records were screened based on the titles and abstracts. At this stage, 384 records were excluded for the following reasons: not relevant to RA or thyroid cancer (n = 312); reviews, comments, or conference abstracts (n = 48); or case reports or case series (n = 24). The full texts of the remaining 68 records were independently screened by two reviewers. A total of 54 studies were excluded at this stage for the following reasons: exclude the literature that does not meet the inclusion criteria (n = 33); outcome was not thyroid cancer-specific (n = 17); did not report HR or SIR with 95% CI, or insufficient data to calculate effect size (n = 4). Finally, fourteen studies (24–37) met the preset inclusion criteria and were included in the meta-analysis (**Figure 1**).

**Table 1** summarizes the characteristics of the fourteen studies included in this meta-analysis. A total of fourteen studies were published between 2009 and 2025. They were conducted in Japan, the United Kingdom, South Korea, China, and the United States. All studies were cohort studies. The combined sample size included 1,970,165 participants, with the sample sizes of each study ranging from 18,850 to 1,313,838. The average age range of the participants was from 52.6 to 64.2 years, and the percentage of female participants ranged from 52.6% to 100%. The average follow-up time was from 3.3 to 17.7 years.

**Table 1:** The basic characteristics of the included literature.

References	Study duration	Country	Study design	Sample size	Sex (male/female)	Mean age(years)	Disease definition	Adjustment for covariates	Mean follow-up(years)	HR/SIR	95% CI
Huang et al., 2014 (36)	From 1996 to 2008	China	Cohort study	30504	6824/23680	53.6	ICD-9	Age	7.4	1.07	0.78-1.46
Zhou et al., 2022 (37)	From January 2006 to April 2015	China	Cohort study	1658	412/1246	57.8	ICD-10	Age, Sex, District of residence	4.09	12.58	7.19-20.43
Chen et al., 2011 (25)	From 1996 to 2007	China	Cohort study	23644	5117/18527	53.08 ± 14.38	ICD-9	Age, Sex, Time	5.90 ± 2.87	1.41	1.36 - 1.47
Cho et al., 2025 (26)	From 2010 to 2017	Korea	Cohort study	245370	61566/183804	57.8 ± 9.4	ICD-10	Age, Sex, RA serological status, Smoking status, Alcohol intake, Physical activity, Income level, BMI, Complications (Diabetes, Hypertension, Dyslipidemia, Chronic kidney disease)	5.5 ± 2.2	0.98	0.85 - 1.13
Choi et al., 2022 (27)	From 2002 to 2015	Korea	Cohort study	15350	4050/11300	58.7	ICD-10	Age, Sex, Income level, Residential area, Obesity, Smoking status, Alcohol intake, Charlson comorbidity index score, Systolic blood pressure, Diastolic blood pressure, Fasting blood glucose, Total cholesterol	6.85	1.01	0.69 - 1.46
Hashimoto et al., 2015 (28)	From 2003 to 2012	Japan	Cohort study	66953	12319/54634	62.7 ± 12.6	ICD-10	Age, Sex	NR	0.59	0.15 - 1.02
Lai et al., 2025 (29)	From 2006 to 2025	China	Cohort study	84477	22917/61560	54	ICD-10	Age, Sex, Cerebrovascular diseases, Chronic kidney diseases, COPD, Ischemic heart diseases, Diabetes, Hyperlipidemia, Hypertension, Hashimoto’s thyroiditis, Graves’ disease, Overweight, Obesity	NR	1.40	1.18 - 1.67
Lee 2019 (24)	From 1996 to 2009	Korea	Cohort study	1885	299/1586	52.6	ICD-10	Age, Sex	5.42	1.75	1.02 - 2.68
Matsui et al., 2025 (30)	From April 2013 to March 2019	Japan	Cohort study	26607	5623/20984	64.16 ± 13.29	/	Age, Sex	3.3	0.76	0.57 - 1.00
Parikh-Patel et al., 2009 (31)	From 1991 to 2002	USA	Cohort study	84475	19239/65236	64.94	ICD-9	Age, Sex, Ethnicity	4.8	0.52(Male)/0.70(Female)	0.11 - 1.52(Male)/0.46 - 1.03(Female)
Park et al., 2024 (32)	From 2009 to 2019	Korea	Cohort study	32656	15468/17188	60.20 ± 9.62	ICD-10	Age, Sex, BMI, Fasting blood glucose level, Smoking status, Alcohol intake, Physical activity, Income level	9.49	1.76	1.07 - 2.90
Yamada et al., 2010 (33)	From April 2001 to October 2005	Japan	Cohort study	7566	1376/6190	55.9 ± 13.3	/	Age, Sex	3.4	1.15	0.31 - 2.95
Yang et al., 2024 (34)	From 1996 to 2018	England	Cohort study	1313838	62681(All Female)	56.6 ± 4.8	ICD-10	Residential area-derived social deprivation, Education, Smoking Status, Alcohol intake, BMI, Strenuous exercise, Age at menopause, Use of menopausal hormone therapy, Duration of past oral contraceptive use, Age at first birth and parity, Age at menarche	17.7 ± 4.9	1.12	0.89 - 1.41
Yu et al., 2016 (35)	From 1997 to 2012	China	Cohort study	35182	8164/27018	52.56 ± 15.53	ICD-9	Age, Sex, Time	7.5	1.24	0.92 - 1.67

BMI, body mass index; COPD, chronic obstructive pulmonary disease; CI, Confidence Interval; ICD, International Classification of Diseases; HR, Hazard Ratio; SIR, Standardized Incidence Ratio.

The diagnostic methods for RA included diagnostic code diagnosis, clinical verification diagnosis, major injury certificate, and clear medication history. The diagnostic methods for thyroid cancer included diagnostic code diagnosis, clinical verification diagnosis, major injury certificate, and patient self-report registration. All studies reported the data after multivariate adjustment, considering the age factor, and some also considered covariates such as gender, body mass index, smoking status, drinking status, physical activity, and socioeconomic factors (**Table 1**). According to the ROBINS-E assessment, there were two low-risk studies, nine medium-risk studies, and three high-risk studies (**Table 2**).

**Table 2:** Summary of bias assessment using the ROBINS-E tool.

Study	D1	D2	D3	D4	D5	D6	D7	Overall
Huang et al., 2014 (36)	Moderate	Low	Low	Low	Low	Low	Low	Moderate
Zhou et al., 2022 (37)	Moderate	Moderate	Low	Low	Moderate	Low	Low	Moderate
Chen et al., 2011 (25)	Moderate	Low	Low	Low	Moderate	Low	Low	Moderate
Cho et al., 2025 (26)	Low	Low	Low	Low	Low	Low	Low	Low
Choi et al., 2022 (27)	Low	Low	Low	Low	Low	Low	Low	Low
Hashimoto et al., 2015 (28)	Moderate	Moderate	Low	Low	Moderate	Moderate	Low	Moderate
Lai et al., 2025 (29)	Moderate	Moderate	Moderate	Low	Moderate	Moderate	Low	Moderate
Lee 2019 (24)	Serious	Serious	Moderate	Low	Moderate	Low	Moderate	Serious
Matsui et al., 2025 (30)	Moderate	Low	Low	Low	Moderate	Low	Low	Moderate
Parikh-Patel et al., 2009 (31)	Serious	Moderate	Low	Low	Low	Low	Moderate	Serious
Park et al., 2024 (32)	Moderate	Low	Low	Low	Moderate	Low	Low	Moderate
Yamada et al., 2010 (33)	Moderate	Moderate	Low	Low	Moderate	Moderate	Low	Moderate
Yang et al., 2024 (34)	Low	Low	Moderate	Low	Low	Low	Low	Moderate
Yu et al., 2016 (35)	Serious	Low	Moderate	Low	Low	Low	Low	Serious

Domains:

D1, Bias due to confounding.

D2, Bias in selection of participants.

D3, Bias in classification of exposures.

D4, Bias due to deviations from intended exposures.

D5, Bias due to missing data.

D6, Bias in measurement of outcomes.

D7, Bias in selection of the reported result.

### 3.2 Meta-analysis

**Figure 2:**
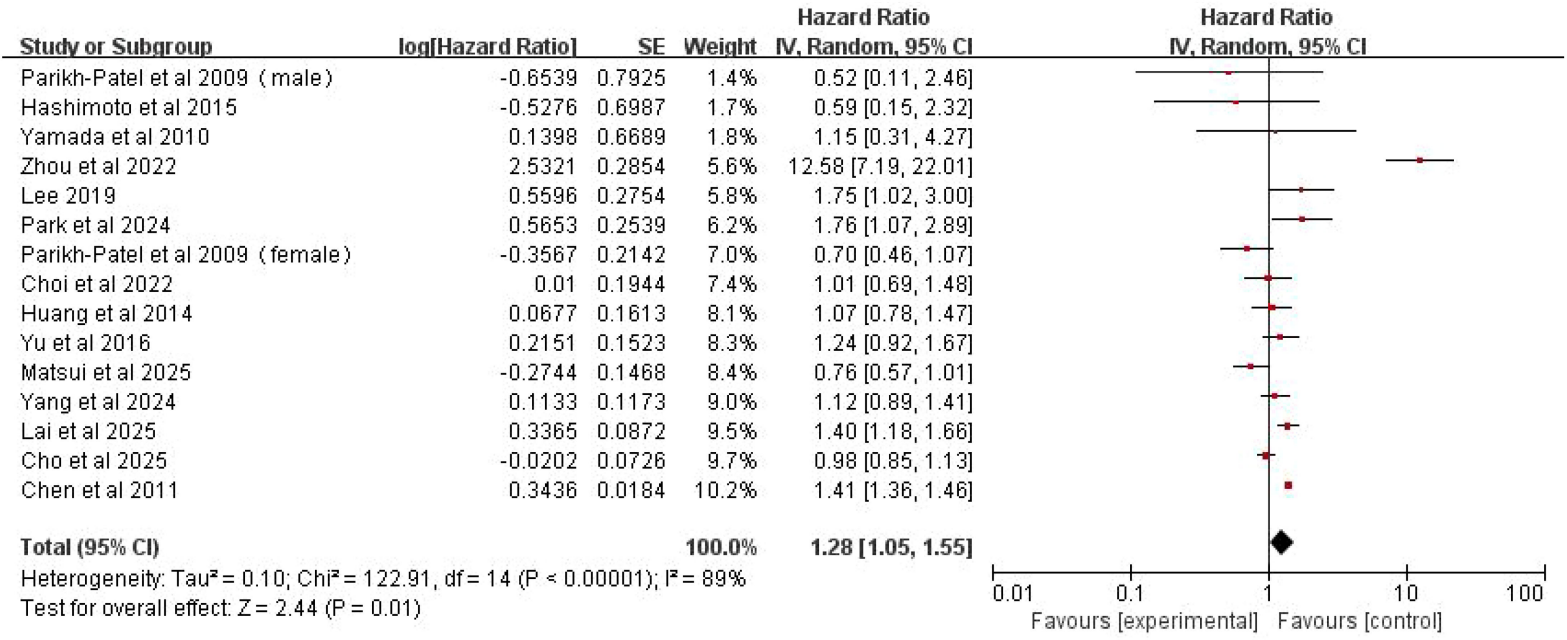
Forest plots of thyroid cancer risk in rheumatoid arthritis.

Fourteen studies have reported the association between RA and the risk of thyroid cancer. Heterogeneity test results: I^2^ = 89%, *P* < 0.00001, using the random effects model. The meta-analysis indicated that the risk of thyroid cancer in patients with RA was higher than that in the general population (HR = 1.28,95%CI (1.05-1.55), *P* = 0.01) (**Figure 2**).

### 3.3 Subgroup analysis

We conducted subgroup analyses based on country (China vs others) and level of covariate adjustment (**Table 3**).

**Table 3:** The pooled results of each subgroup.

Subgroup analysis	No. of studies	Heterogeneity		HR (95% CI)	*P*-value
		I*^2^*	P		
Country					
China	5	94%	< 0.00001	1.79(1.26-2.54)	0.001
Others	9	49%	0.04	1.01(0.85-1.21)	0.88
Level of covariate adjustment					
Parsimonious (age and/or sex only)	5	52%	0.08	1.03(0.73-1.44)	0.88
Extended (age, sex, + ≥1 covariate)	9	91%	< 0.00001	1.38(1.09-1.73)	0.006
Effect size					
HR	5	70%	0.01	1.18(0.97-1.43)	0.09
SIR	9	90%	< 0.00001	1.34(0.93-1.93)	0.12

Among the 5 studies conducted in China, the pooled HR was 1.79 (95% CI: 1.26-2.54, *P* = 0.001); among the 9 studies in other countries (Japan, Korea, the United States, and the United Kingdom), the pooled HR was 1.01 (95% CI: 0.85-1.21, *P* = 0.88).

In the parsimonious adjustment group (5 studies), the pooled HR was 1.03 (95% CI: 0.73-1.44, *P* = 0.88); in the extended adjustment group (9 studies), the pooled HR was 1.38 (95% CI: 1.09-1.73, *P* = 0.006).

### 3.4 Sensitivity analysis

**Figure 3:**
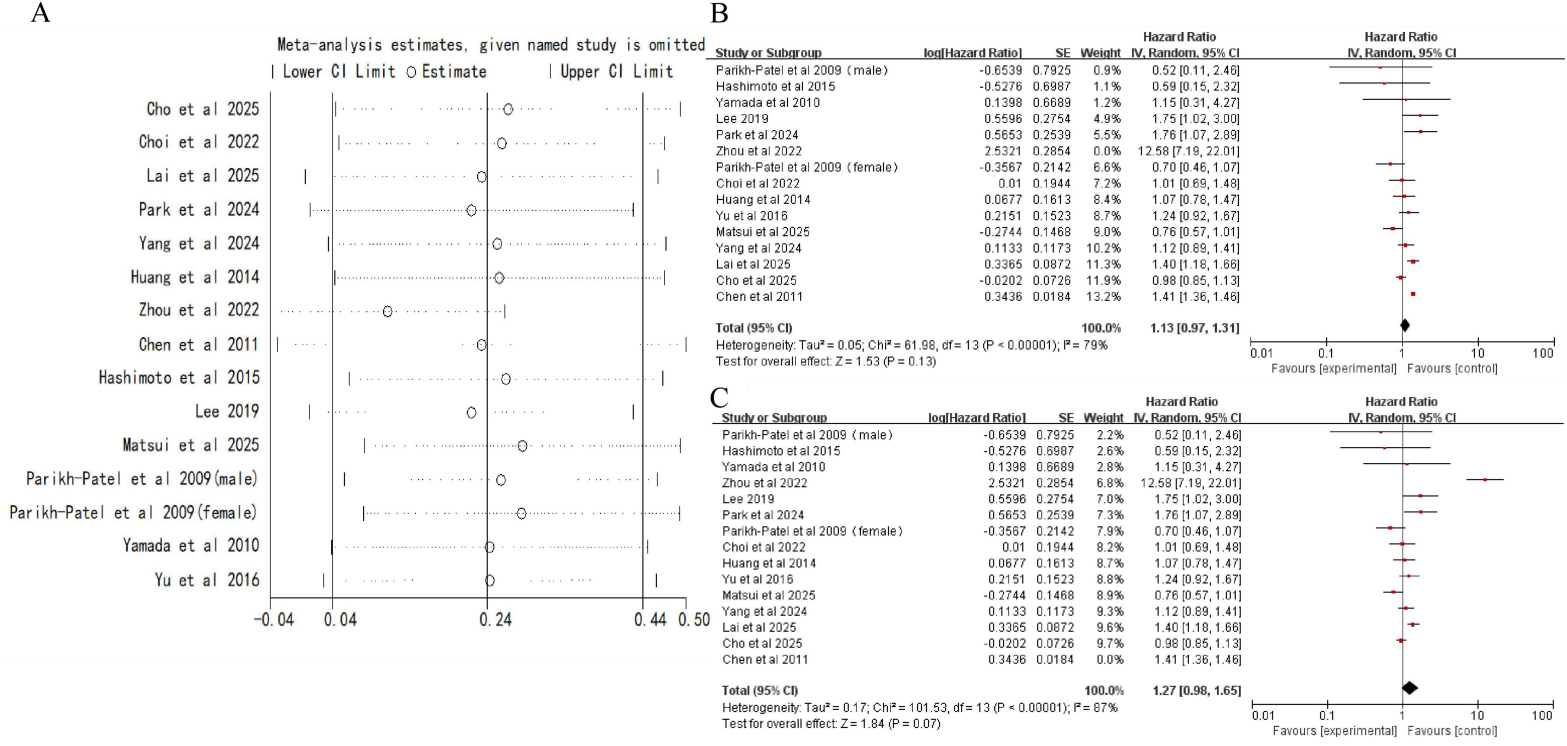
The results of the sensitivity analysis. **(A)** the method of elimination one by one; **(B)** forest plots of thyroid cancer risk in rheumatoid arthritis after excluding the study by Zhou et al., 2022 (37); **(C)** forest plots of thyroid cancer risk in rheumatoid arthritis after excluding the study by Chen et al., 2011 (25).

By eliminating each study one by one and then conducting a meta-analysis anew, the stability of the test results was examined. We found that after excluding the studies by Zhou et al. (35) or Chen et al. (23), the statistical significance of the combined effect size disappeared (*P* > 0.05) (**Figure 3**). This indicated that the overall effect observed may not be robust. To further assess the robustness of pooling different effect measures, we performed separate meta-analyses for effect sizes reported as HR and those reported as SIR. Among the 15 effect sizes extracted from 14 included studies (one study reported sex-stratified HRs), 5 were HRs and 10 were SIRs. The pooled HR from HR-only effect sizes was 1.18 (95% CI: 0.97-1.43, P = 0.09, I² = 70%), while the pooled SIR from SIR-only effect sizes was 1.34 (95% CI: 0.93-1.93, P = 0.12, I² = 90%). Neither estimate reached statistical significance. The lack of statistical significance in both separate analyses supports the view that the significant result in the primary pooled analysis (HR = 1.28, 95% CI: 1.05-1.55, P = 0.01) is not robust and may be influenced by the pooling of different effect measures or by specific high-weight studies.

### 3.5 Publication bias

**Figure 4:**
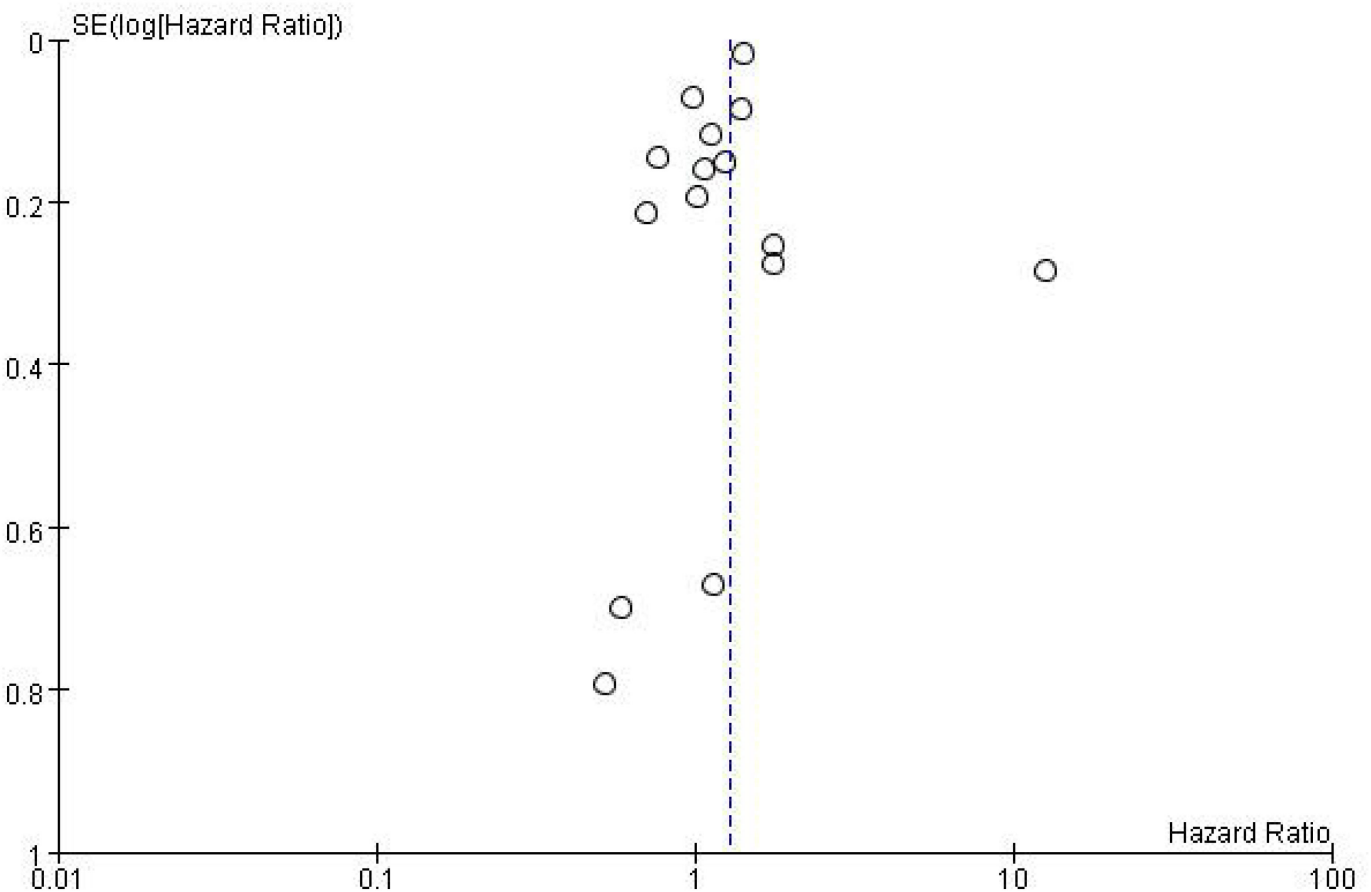
Funnel plots of thyroid cancer risk in rheumatoid arthritis.

The funnel plot showed that the data points were approximately symmetrically distributed, indicating a relatively low possibility of publication bias. Begg’s test (*P* = 0.624) and Egger’s test (*P* = 0.714) indicated no significant bias (**Figure 4**).

## 4 Discussion

This study included fourteen articles, involving a total sample size of 1,970,165 participants. It comprehensively and systematically compared the association between RA and the risk of thyroid cancer. The meta-analysis indicated that, in the overall analysis, the risk of thyroid cancer in RA patients was higher than that in the general population. This finding was consistent with the overall trend of increased risk of malignant tumors in RA patients in previous studies (10). Several early large-scale meta-analyses reported an increased risk of hematological malignancies and lung cancer in patients with RA, while the risks of colorectal cancer and breast cancer decreased (10). However, less attention was paid to thyroid cancer. This study found that the association between RA and thyroid cancer was statistically significant, suggesting that thyroid cancer should be taken into consideration when assessing the tumor risk of RA patients. The study conducted by Lai et al (29) was based on a multinational, multi-institutional electronic medical record database. In this large-scale real-world cohort study, the results were consistent with ours. The current evidence supports a positive association between RA and the risk of thyroid cancer.

The subgroup analysis revealed a stronger association between RA and thyroid cancer in the Chinese population, while no significant risk was observed in other countries. This may reflect differences in genetic background, environmental exposures, and screening intensity. Thyroid cancer incidence in China is heavily influenced by overdiagnosis, so the elevated risk in Chinese patients may reflect more intensive surveillance rather than a genuine biological increase (38, 39). RA patients undergo more frequent thyroid ultrasound examinations due to comorbidity screening. China has a history of iodine deficiency and implemented universal salt iodization; both insufficient and excessive iodine intake increase thyroid disease risk (40, 41). Chinese patients with RA may be more prone to thyroid cancer under the background of RA’s immune disorder due to their specific iodine nutritional status. China’s clinical practice relies more on conventional synthetic DMARDs (csDMARDs), while biologics and targeted agents are less accessible due to cost and insurance limitations, potentially leading to suboptimal disease control and higher thyroid cancer risk (42). In contrast, developed countries have stricter oversight of over-screening, earlier and more widespread use of biologics and targeted DMARDs, and more robust insurance coverage, resulting in higher RA remission rates (43, 44).

The subgroup analysis stratified by adjustment for age and/or sex only yielded a non-significant result (HR = 1.03), whereas studies with more comprehensive confounder adjustment showed a significant association (HR = 1.38). This pattern indicates that residual confounders remain a major concern. Important factors such as BMI, smoking, iodine intake, thyroid screening intensity, and RA-related medications were not adequately controlled in studies limited to age and sex adjustment. These factors are known to be associated with both RA and thyroid cancer and may distort the true association. In addition, the high heterogeneity (I² = 91%) in the fully adjusted subgroup suggests that differences in study populations, screening practices, thyroid cancer diagnostic intensity, and regional iodine status may still contribute to variability in effect sizes. Notably, the loss of significance in the age–sex–only subgroup supports the notion that the observed association between RA and thyroid cancer is sensitive to confounder adjustment and should be interpreted cautiously.

Regarding the pooling of HR and SIR, we acknowledge that combining different effect measures requires careful justification. Under the rare disease assumption, HR and SIR are considered approximately equivalent as both estimate relative risk when the outcome event rate is low (20–23). However, to test the validity of this assumption, we conducted separate meta-analyses for HR-only and SIR-only effect sizes. Among the 15 effect sizes extracted from 14 included studies (one study reported sex-stratified HRs), the HR-only analysis (n = 5 effect sizes) yielded a pooled estimate of 1.18 (95% CI: 0.97-1.43, P = 0.09, I² = 70%), and the SIR-only analysis (n = 10 effect sizes) yielded 1.34 (95% CI: 0.93-1.93, P = 0.12, I² = 90%). Neither reached statistical significance, contrasting with the primary pooled analysis (P = 0.01). This discrepancy suggests that the statistically significant result in the primary analysis may be driven by the pooling of different effect measures or by the differential weighting of studies reporting HR versus SIR. Notably, the SIR-only analysis showed very high heterogeneity (I² = 90%), indicating substantial variability among studies using this measure, which further limits confidence in the pooled estimate. Taken together, these sensitivity analyses indicate that the association between RA and thyroid cancer risk is not robust and should be interpreted with caution.

When the studies by Zhou et al. (37) or Chen et al. (25) were excluded, the statistical significance of the pooled effect size disappeared. Chen et al.’s (25) study has an extremely large sample size, and in all the included studies, its effect size and accuracy determined that it would have a very high statistical weight in the combined analysis. This study used records from the major injury database to identify the diagnosis of RA and thyroid cancer. This database adopted strict and clear inclusion criteria, ensuring the accuracy of all the diagnosis conclusions and the high reliability of the data. Additionally, with a large sample size, it may result in a narrower confidence interval. Moreover, most RA patients in this study received regular physical and laboratory evaluations during the observation period, and there was no delay in the diagnosis of thyroid cancer. The monitoring bias may have led to a partial increase in the cancer incidence among these patients. Zhou et al.’s (37) research was conducted on inpatients from large tertiary hospitals in China. They implemented highly standardized long-term management for patients with rheumatoid arthritis, including systematic screening for comorbidities and structured follow-ups. Therefore, more frequent and comprehensive examinations might be conducted during the follow-up, including thyroid ultrasound and functional tests. The diagnosis of thyroid cancer in this study also had no delay, which might lead to a higher cancer detection rate.

From the perspective of the autoimmune mechanism, RA is a typical autoimmune disease. The immune system becomes abnormally activated, generating a large number of autoantibodies, which then attack the body’s own tissues and organs (45). The thyroid gland, as an important endocrine organ in the human body, has multiple antigens on its cell surface. In the state of immune disorder in RA patients, these antigens may be wrongly recognized by the immune system as foreign antigens, thereby triggering an immune response and causing damage to the thyroid tissue (46). Long-term immune attacks may potentially contribute to genetic alterations in thyroid cells, which could in turn be associated with elevated thyroid cancer risk (47). Studies (45) have shown that in patients with RA, the functions of T lymphocytes and B lymphocytes are dysregulated. T lymphocytes are abnormally activated and secrete various inflammatory cytokines, such as tumor necrosis factor-α and interleukin-6. These cytokines not only participate in the inflammatory process of RA but may also be implicated in the growth, proliferation, and differentiation of thyroid cells. The B lymphocytes become over-activated and produce many autoantibodies, such as anti-thyroid peroxidase antibody (TPOAb), anti-thyroid globulin antibody (TgAb), etc (46). These antibodies bind to thyroid tissue, activating the complement system and causing cellular damage, which may create a favorable microenvironment for thyroid tumorigenesis (47).

This study has certain limitations. Firstly, high heterogeneity (I² = 89%) was detected in the main analysis. Potential causes include regional differences in thyroid screening intensity, inconsistent diagnostic criteria, variations in population characteristics, differences in iodine intake, disparate RA treatment patterns, and unequal adjustment for confounders. These factors together lead to significant between-study inconsistency and limit the robustness of the pooled results. Secondly, the association was significantly affected by the extent of confounding adjustment. The pooled effect became non-significant among studies with parsimonious adjustment (age and/or sex only) but remained significant in studies with extended confounder adjustment. This pattern suggests that residual confounding remains a major concern, as important factors such as BMI, smoking, iodine intake, thyroid screening intensity, and RA-related medications were not adequately controlled in minimally adjusted studies. Therefore, residual confounding may partially explain the observed increased risk. Finally, all the included studies were observational designs and cannot establish causal relationships. They can only suggest associations.

## 5 Conclusions

This meta-analysis suggests that RA may be associated with an increased risk of thyroid cancer, with elevated risk observed in the Chinese population and in studies with more comprehensive covariate adjustment. However, these findings are not robust in sensitivity analyses. Furthermore, the difference between subgroups is not statistically significant. Given limitations due to high heterogeneity and potential confounding, well-designed prospective studies are needed to further verify this association.

## Data Availability

The original contributions presented in the study are included in the article/**Supplementary Material**. Further inquiries can be directed to the corresponding authors.

## Supplementary material

Supplementary Table 1 Search strategies.
