# Supplementary material for: Meta-analysis and systematic review: the association between rheumatoid arthritis and the risk of thyroid cancer
Source: Front Immunol. 2026 Jun 3;17:1769646. doi: 10.3389/fimmu.2026.1769646 (PMC13273124; doi:10.3389/fimmu.2026.1769646)
Supplement: Supplementary Table 1 — Search strategies. [file Table1.pdf]

Table S1 Search strategies

Number	Search words
#1	Thyroid Neoplasms [MeSH Terms]
#2	Neoplasm, Thyroid [Title/Abstract]
#3	Thyroid Neoplasm [Title/Abstract]
#4	Neoplasms, Thyroid [Title/Abstract]
#5	Thyroid Carcinoma [Title/Abstract]
#6	Carcinomas, Thyroid [Title/Abstract]
#7	Carcinoma, Thyroid [Title/Abstract]
#8	Thyroid Carcinomas [Title/Abstract]
#9	Cancer of the Thyroid [Title/Abstract]
#10	Cancer of Thyroid [Title/Abstract]
#11	Thyroid Cancers [Title/Abstract]
#12	Thyroid Cancer [Title/Abstract]
#13	Cancers, Thyroid [Title/Abstract]
#14	Cancer, Thyroid [Title/Abstract]
#15	Thyroid Adenoma [Title/Abstract]
#16	Adenomas, Thyroid [Title/Abstract]
#17	Adenoma, Thyroid [Title/Abstract]
#18	Thyroid Adenomas [Title/Abstract]
#19	Cancer, Thyroid [Title/Abstract]
#20	Thyroid Adenoma [Title/Abstract]
#21	OR/1-20
#22	Arthritis, Rheumatoid [MeSH Terms]
#23	Rheumatoid Arthritis [Title/Abstract]
#24	OR/22-23
#25	#21AND#24
